# The incidence of unexpected uterine malignancies in hysterectomies carried out for benign indications

**DOI:** 10.1007/s00432-022-04343-0

**Published:** 2022-09-09

**Authors:** Yuanyuan Ding, Yana Han, Sanyuan Zhang, Xiaorong Shi

**Affiliations:** Department of Gynecology, First Hospital of Shanxi Medical University, Taiyuan, Shanxi China

**Keywords:** Benign indication, Hysterectomy, Unexpected gynaecological malignancy

## Abstract

**Purpose:**

The aim of the present study was to evaluate the incidence of unexpected uterine malignancies in patients undergoing hysterectomy for benign indications and to evaluate their clinical characteristics.

**Methods:**

We conducted a retrospective review of patients who underwent benign hysterectomy in the Department of Gynecology, the First Hospital of Shanxi Medical University from January 2015 to December 2020. The clinical data of these patients were retrieved and collected.

**Results:**

Their median age was 49.8 years (31–82 years). The mean parity was 1.86 ± 2.54. Their mean BMI was 27.5 ± 7.6 kg/m2. 42.90% were (2438/5683) postmenopausal. The benign indications of procedure were as follows: symptomatic uterine leiomyomas 2218/5683 (39.02%), pelvic organ prolapse 1406/5683 (24.74%), symptomatic endometriosis or adenomyosis 1132/5683 (19.91%), and 927/5683 (16.31%) to treat other benign conditions such as abnormal uterine bleeding, infection, polyps, and endometrial hyperplasia without atypia. In minimally invasive surgery subgroups, 1560/2621 (59.52%) specimens were removed by in-bag manual morcellation through vaginal cuff. The mean operative time of minimally invasive surgery with in-bag morcellation was shorter than abdominal hysterectomy (96.75 ± 35.7 vs. 140 ± 32.6, *P* < .001), and the estimated blood loss was also less than abdominal hysterectomy (47.35 ± 42.3 vs. 170 ± 60.4, *P* < .001). A total of 19/5683 (0.33%) unexpected uterine malignancies were recorded, of which 14/5683 (0.26%) were unexpected endometrial carcinomas and 5/5683 (0.08%) were unexpected uterine sarcomas.

**Conclusion:**

Preoperative examination in the context of benign hysterectomy must be undertaken with care, and patients should be educated about the very slight possibility of a malignant diagnosis.

## Introduction

Hysterectomy is the most commonly performed gynecologic procedure for benign conditions, such as leiomyoma, abnormal bleeding, endometriosis, endometrial polyps, and pelvic organ prolapse, with approximately 600,000 performed annually in the United States (Multinu et al. 2019), 100,000 in the United Kingdom and 70,000 in France (Ouldamer et al. 2014).

Routine examinations prior to hysterectomy include gathering information about medical history, gynecological examination, ultrasound, and sometimes Magnetic Resonance Imaging (MRI) to exclude a potential uterine malignancy. Transvaginal ultrasound is a valid tool in identifying patients with a high risk of endometrial cancer (EC) and MRI may be useful in differentiating uterine sarcoma (US). Additional invasive endometrial biopsy is used for confirmation of diagnosis in suspicious cases. However, some studies have demonstrated that the endometrial sampling in identifying EC and US to be less precise than previously thought (Bansal et al. 2008; Visser et al. 2017). Up to 0.5% of the surgically resected as presumably benign myomas will be diagnosed as sarcomas at histopathologic analysis (RJ. M. 2020).

Therefore, unexpected uterine malignancies seem inevitable. In the studies from some different countries investigating the incidence of unexpected uterine malignancy after hysterectomies for benign indications, reported that the incidence of endometrial carcinoma was 0.12–1.02%, and uterine sarcoma 0.06–0.22% (Mahnert et al. 2015; Yuk et al. 2016; Desai et al. 2019; Wagner et al. 2019). However, they vary widely between countries.

Until now, the epidemiology of hysterectomy and the incidence of unexpected uterine malignancies in hysterectomies for presumed benign disease in China, remain largely unknown. The aims of our study were to evaluate the incidence, characteristics, preoperative examinations and outcomes of patients with unexpected uterine malignancies found incidentally after hysterectomies for presumed benign disease.

## Materials and methods

This retrospective analysis included patients treated with benign hysterectomies by any approach (abdominal, vaginal, laparoscopic or robot-assisted) in the department of Gynecology of the First Hospital of ShanXi Medical University from January 2015 to December 2020. Previous studies almost always have included cases of before 2014, the year of FDA warning. However, surgical procedures have changed since then, we chose patients from a later period of that time, and which may be more in line with present or future conditions. Patients with an unexpected diagnosis of EC or US within the corpus uteri confirmed by final pathological report were selected from the chart review.

Patients with a preoperative surgical indication of cancer, cervical dysplasia cases whose cervical biopsy result was cervical intraepithelial neoplasia 2 (HSIL) or higher, or endometrial hyperplasia with atypia were excluded, as they were often suspected of having cancer. Cases with an obstetric indication for hysterectomy and those with missing data for preoperative indication were also excluded from this analysis.

Medical records of these patients were retrieved to collect clinical data (age, Body Mass Index, parity, menstrual status, the primary preoperative symptoms, and pathologic data). Special attention was given to the mode of hysterectomy, specimen removal, operative time, and intraoperative blood loss. The course of preoperative workup, FIGO stage, further treatments, and survival data of UUM were also recorded.

Women were considered postmenopausal if their last menstrual period occurred > 1 year before the preoperative visit.

Our policy requires results of Thin-prep cytology test as well as High-risk human papillomavirus test within 1 years of surgery and pelvic ultrasound examination within 3 months. In postmenopausal women, a cutoff value of 4 mm for endometrial thickness, and in women of reproductive age, the cutoff value was 15 mm. Patients with symptoms of vaginal abnormal bleeding and/or a thicker endometrium and/or other endometrial abnormalities (such as heterogeneous endometrium, polyps, etc.) suggested by ultrasound were accepted hysteroscopy. And further endometrial biopsy [dilation and curettage (D&C), or hysteroscopic biopsy or both] was done if the physician who performed the hysteroscopy deems it necessary. During the minimally invasive surgery (MIS, laparoscopic hysterectomy and robot-assisted hysterectomy), all the specimens were removed through vaginal cuff. If the specimens were too large, we removed them after manual morcellation in the bag to avoid the spread of cells and tissues. If an unexpected uterine malignancy was detected after primary surgery, a full radiographic evaluation will be conducted and we performed re-operation or other treatments according to the guidelines often within 1 month.

Values are expressed as mean ± SD. Numeric data were analyzed using the t test if normally distributed, and if not, the Mann–Whitney test was used for comparison of data.

## Results

### Study cohort

We identified 5683 patients who were treated for benign gynecological conditions with hysterectomy in the specified period of time. Their median age was 49.8 ± 12.53 years (31–82 years). The mean parity was 1.86 ± 2.54. Their mean BMI was 27.5 ± 7.6 kg/m2. 42.90% were (2438/5683) postmenopausal. Indications for elective surgery were: symptomatic uterine leiomyomas 2218/5683 (39.02%), pelvic organ prolapse 1406/5683 (24.74%), symptomatic endometriosis or adenomyosis 1132/5683 (19.91%), and 927/5683 (16.31%) to treat other benign conditions such as abnormal uterine bleeding, infection, polyps, and endometrial hyperplasia without atypia. The 1527/5683 (26.87%) were vaginal hysterectomies, 2486/5683 (43.74%) were total laparoscopic hysterectomies, 1172/5683 (20.62%) were abdominal hysterectomies, 363/5683 (6.38%) were abdominal supracervical hysterectomies, and 135/5683 (2.37%) were robot-assisted hysterectomies. A total of 19/5683 (0.33%) unexpected uterine malignancies were recorded, of which 14/5683 (0.26%) were unexpected EC and 5/5683 (0.08%) were unexpected US. The demographic and clinical characteristics of patients are summarized in Table 1.

**Table 1:** The demographic and clinical characteristics of patients

Parameter	*n* (%)
Age, mean	49.8 ± 12.53
Menstrual status	
Postmenopausal status	2438 (42.90)
Premenopausal status	3245 (57.10)
BMI, mean	27.5 ± 7.6
Parity, mean	1.86 ± 2.54
Surgical indication	
Symptomatic uterine leiomyomas	2218 (42.10)
Pelvic organ prolapse	1406 (15.78)
Symptomatic endometriosis or adenomyosis	1132 (10.52)
other benign conditions	927 (78.94)
Surgical technique	
Laparotomy	1535 (27.01)
Laparoscopy	2486 (43.74)
robot-assisted hysterectomy	135 (2.37)
Vaginal hysterectomy	1527 (26.87)
Pathology result	
Benign pathology reports	5664 (99.67%)
Unexpected endometrial carcinomas	14 (0.24%)
Unexpected uterine sarcomas	5 (0.08%)
Method of specimen removal	
MIS with in-bag manual morcellation	1560 (59.52%)
MIS without in-bag manual morcellation	1061 (40.48%)

*BMI* Body mass index, *MIS* Minimally invasive surgery

Subgroup analysis depending on surgical approach showed unexpected uterine malignancies to be most frequently diagnosed within the laparoscopic hysterectomy subgroup, with incidence rates of 0.44%. In MIS subgroup, 1560/2621 (59.52%) specimens were removed by in-bag manual morcellation through vaginal cuff. The postoperative data of the study subgroup are summarized in Table 2. As two alternatives to MIS, the mean operative time of subgroup of MIS with in-bag morcellation was shorter than subgroup of abdominal hysterectomy (96.75 ± 35.7 vs. 140 ± 32.6, *P* < 0.001), and the estimated blood loss was also less than the subgroup of abdominal hysterectomy (47.35 ± 42.3 vs. 170 ± 60.4, *P* < 0.001).

**Table 2:** Postoperative data of the study subgroups

Parameter	MIS with in-bag morcellation (*n* = 1560)	TAH (*n* = 1172)	*P* value
Mean operative time (min)	96.75 ± 35.7	140 ± 32.6	< 0.001
Estimated blood loss (ml)	47.35 ± 42.3	170 ± 60.4	< 0.001

### Unexpected endometrial carcinomas

In total, 12/14 (85.7%) tumors were FIGO stage IA, 1/14 (7.1%) was FIGO stage IB and 1/14 (7.1%) was FIGO stage III disease. 11/14 (71.4%) carcinomas were G1, 2/14 (14.2%) were G2 and 1/14 (7.1%) was G3. Endometrioid histotype (Type I) was present in 11/14 (71.4%) tumors, 3/14 (21.4%) tumors were of serous histotype (Type II). The molecular classes of endometrial cancer were unclear as gene sequencing has not been carried out in our institution. According to current treatment guidelines, 8/14 (57.1%) were re-explored to complete staging or remove both ovaries (as salpingectomy was accompanied hysterectomy in premenopausal women), adjuvant therapy was recommended in 5/14 (35.7%) cases. During preoperative workup, 6/14 (42.8%) patients with unexpected EC received D&C, 2/14 (14.2%) received hysteroscopic biopsy, 2/14 (14.2%) received hysteroscopy only, and 2/14 (14.2%) received both of them (D&C was performed if hysteroscopic biopsy is insufficient). The remaining 2/14 (14.2%) did not undergo invasive examination because their ultrasound did not indicate endometrial abnormalities. Initial symptoms included postmenopausal uterine bleeding or menstrual disorders in 11/14 cases (71.4%) cases, among them, 1/11 combined with endometrial polyps, 3/11 with fibroids and 2/11 with adenomyoma. Of the remaining three, two were due to pelvic organ prolapse, and one was due to polyps without bleeding. Disease relapse was observed in 2/14 (14.2%) patients with unexpected EC, 11 and 23 months after initial diagnosis, respectively. In these cases, serous type histology and poorly differentiation were present, and the one diagnosed with FIGO stage III, died after second-line palliative chemotherapy. Median follow-up for unexpected EC patients was 32 months (16–70 months). Details of clinicopathological data of unexpected EC are given in Table 3A.

**Table 3:** Clinicopathological characteristics of patients with unexpected malignant uterus pathology (unexpected EC and unexpected US)

Parameter	#/Number of patients
A	
Unexpected endometrial carcinoma	14
Histology	
Endometrioid	11
Serous	3
FIGO stage	
IA	12
IB	1
III	1
Tumor grade	
Grade 1	11
Grade 2	2
Grade 3	1
Initial symptoms	
Abnormal bleeding	11
Pelvic organ prolapse	2
Polyps	1
Preoperative evaluation	
D&C	6
Hysteroscopy ± biopsy	4
Both	2
Re-operation	
Complete staging	4
Remove ovaries	4
Follow-up	
Relapse of disease	2
Disease specific death	1
B	
Unexpected uterine sarcoma	5
Type	
Leiomyosarcoma	2
Endometrial stromal sarcoma (low-grade)	3
FIGO stage	
IA	2
IB	3
Preoperative evaluation	
Hysteroscopy ± biopsy	1
Both	1
Re-operation	
Complete staging	1
Remove ovaries	2
Follow-up	
Relapse of disease	2
Disease specific deaths	1

A total of ten unexpected EC patients were primarily treated with laparoscopic hysterectomy, and 3/10 (30.0%) underwent in-bag manual morcellation through vaginal cuff. All three patients were re-explored to complete the staging operation (1/3) or remove both ovaries (2/3). None were identified as having disseminated disease during surgical re-exploration, and no relapse during the follow-up.

### Unexpected uterine sarcomas

2/5 (40%) were confirmed to be leiomyosarcomas (LMS) and 3/5 (60%) were confirmed as low-grade endometrial stromal sarcoma (LGESS). Most unexpected US were early stage, with 2/5 (40%) and 3/5 (60%) being stage IA (< 5 cm) and stage IB (> 5 cm), respectively. Poor differentiation (grade 3) was present in 1/5 (20%) tumors. Further adjuvant treatment regiments were decided upon after complete surgical tumor staging. A total of 2/5 (40%) patients received adjuvant therapy. Disease recurrence occurred in 2/5 (40%) of patients diagnosed with leiomyosarcomas. These patients presented with pulmonary metastasis and peritoneal recurrence. In all cases, recurrence of disease was treated with palliative chemotherapy and 1 case was dead 3 months after recurrence. Median follow-up for unexpected US patients was 24 months (13–42 months). Details of clinicopathological data of unexpected US are given in Table 3B.

Among preoperative examination, 3/5 (60%) patients had multiple uterine fibroids with a maximum diameter of more than 5 cm. 2/5 (40%) had symptoms of abnormal bleeding, all of which were characterized by increased menstruation. One of them (20%) received hysteroscopic biopsy and the other (20%) received both hysteroscopic biopsy and D&C. 2/5 (40%) had no symptom, and 1/5 (20%) was operated on for prolapse. On ultrasound, 5/5 (100%) showed that the echo of uterine myoma was inhomogeneous and neither rich in blood flow signal nor low resistance indices. 2/5 (40%) indicated cystic degeneration, and 1/5 (20%) suggested red degeneration. Only one patient with accidental sarcoma was treated mainly by laparoscopic hysterectomy. Due to the small size of the tumor, direct removal was chosen instead of manual morcellation in the bag.

## Discussion

In our study, 19/5683 (0.33%) patients were diagnosed with UUM after benign hysterectomy, approximately one in every 300 patients. 0.26% were unexpected EC, 0.08% were unexpected US (Table 1). These results are similar to those of Wagner et al. (2019). Incidence of unexpected EC and unexpected US vary widely between countries from 0.12% and 0.06% in South Korea to 1.02 and 0.22% in United States, respectively (Mahnert et al. 2015). However, the lower incidence rates are probably due to lower crude incidence rate of endometrial cancer in South Korea (8.7/100,000 in South Korea vs. 19/100,000 in the United States) (Yuk et al. 2016; Lortet-Tieulent et al. 2018).

It seems that in cases of unexpected uterine malignancies, tumors tend to be at early stage. Wagner et al. reported 45 cases of unexpected uterine malignancies in 10,756 patients. Among them, 40/45 were stage FIGO I (Wagner et al. 2019). These results are in line with (Yildiz et al. 2021). In this study, 18/19 were FIGO stage I, only 1/19 were FIGO stage III, of whom histopathologic diagnosis was serous endometrial carcinoma with pelvic lymph node metastasis.

Abnormal bleeding were still the most common symptom of unexpected uterine malignancies. Among 19 cases of unexpected uterine malignancies in our results, 13/19 (68.42%) represented with abnormal bleeding, especially in unexpected EC with an incidence of 11/14 (78.57%). Genc et al. found the incidence of EC in postmenopausal women with no bleeding was 0.5 vs. 17.6% with bleeding (Genc et al. 2015). As the incidence of uterine cancer increases with age, and over 90% occurring in women over 50 years, more attention should be paid to women with perimenopausal or postmenopausal bleeding.

Transvaginal ultrasound (TVS) is usually the first step of evaluation. In postmenopausal women, an endometrial thickness (ET) cutoff of 4 mm has a sensitivity of 98%, with a specificity ranging from 36 to 68% (Gentry-Maharaj 2020). In premenopausal women, cut-offs vary dependent on menopausal status. Tsuda et al. reported the endometrial thickness has a range from 5.4 mm to further 11.1 mm through a menstrual cycle (Tsuda et al. 2018). Although simple measurement of endometrial thickness has a limited role in this population, ultrasonography may nonetheless be clinically useful by adding further characterization of the endometrial lining such as heterogeneity or cystic changes increases the diagnostic ability (Kim et al. 2016). However, a thickened endometrium is more indicative of Type I EC risk, whereas Type II ECs are more often associated with an atrophied endometrium (Felix et al. 2010).

During preoperative workup, 12/19 (63.2%) patients with UUM received endometrial sampling (D&C or hysteroscopic biopsy), 2/19 (10.5%) received hysteroscopy without biopsy, as they were not suspected of being malignant during the inspection, and 5/19 (26.3%) did not undergo invasive examination because their ultrasound did not indicate endometrial abnormalities (Table 3). Endometrial sampling, used almost uniformly for the preoperative diagnosis of EC, which can be performed by office endometrial biopsy, hysteroscopic biopsy, or D&C. However, sampling insufficiency remains problems that need to be solved for effective screening, even curetting can only evaluate less than half of the uterine cavity in approximately 60% of D&C procedures, which can result in false-negative diagnoses (Goldstein et al. 1997).

Hysteroscopic biopsy performs biopsy under direct visualization guide, which is over blind biopsy techniques, and has been widely accepted as the gold standard for the investigation of endometrial pathology (Carugno et al. 2021). Nicole analyzed 45 related literatures and found hysteroscopic biopsies show a higher agreement (89%) compared with D&C (70%) in grade diagnosis of EC(4). Gkrozou et al. (2015) in a meta-analysis over 9000 patients found a high diagnostic accuracy of hysteroscopy for endometrial cancer diagnosis (estimated sensitivity 82.6% and specificity 99.7%) (Gkrozou et al. 2015). However, data on hysteroscopic procedures are difficult to analyze as hysteroscopy is a subjective diagnostic test depending on the experience, the knowledge and the capability of the performing physician as well as on menopausal status of the patient (Gkrozou et al. 2015).

Up to now, the major clinical challenge in the management of uterine sarcomas remains early diagnosis. As symptoms are unspecific, and lack of pathognonomic features, most cases are diagnosed as incidental findings after hysterectomy or morcellation of fibroids (P. M. 2018). According to previous reports, the diagnostic sensitivity of ultrasound for US was just 11.0% (Li et al. 2020), and the sensitivity of preoperative sampling is discrepant from 35.3 to 64% (Bansal et al. 2008; Hinchcliff et al. 2016). In our study, 2/5 cases of unexpected US underwent D&C and hysteroscopic biopsy, respectively, and found no malignant pathological features. However, a preoperative biopsy is technically not always possible. As US is mostly located in the myometium, and biopsy maybe less accessible by standard endometrial sampling techniques (Bansal et al. 2008).

There is 2621/5683 (46.1%) women prefer MIS in this study. Compared with laparotomy, MIS is associated with significantly lower intraoperative blood loss, fewer wound complications, and shorter hospital stay (Vargas et al. 2015). In addition, shorter surgical time were also observed in our study. The limitation of removing large specimens through port site of MIS makes the morcellation indispensable. However, performing intracorporeal morcellation may lead to a scattering of benign and malignant tissues, and cause a poorer prognosis by upstaging the occult cancer (Zullo et al. 2020). After the US Food and Drug Administration (FDA) warning in April 2014 (Solima et al. 2015), there was a subsequent shift of management strategies from the use of MIS to the use of open abdominal procedures, along with significantly more major and minor complications (Multinu et al. 2018). In order to maintain the benefits of MIS, the method of using a power morcellator or manual extraction to remove the specimen from inside of the bag has been more and more prevalent (Devassy et al. 2019; Weng et al. 2018).

In MIS subgroup of present study, 1560/2621 (59.5%) specimens were removed by in-bag manual morcellation through vaginal cuff as they were too large for being pulled through the vagina entirely. The mean operative time of MIS with in-bag morcellation was shorter than subgroup of abdominal hysterectomy (*P* < 0.001). And the estimated blood loss was also less than the subgroup of abdominal hysterectomy (*P* < 0.001).

Although in-bag morcellation may appear to be a tempting solution, there are limited data on the effectiveness and safety of in-bag morcellation compared to uncontained power morcellation (Zullo et al. 2020). And the high incidence of bag rupture (33.3%) (Solima et al. 2015) and the leakage through lateral port in multiport surgery (Cohen et al. 2016) are still questioning the safety of this method. However, Emery et al. performed a leak test of a specially designed endoscopic bag system, and suggested that cell diffusion occurred before morcellation, and even in cases of open surgery and intact specimen extirpation, there may be tissue disruption and fluid spread (Lambat Emery et al. 2019). In conclusion, more research is needed to confirm or innovate feasible improvements.

## Conclusion

In present study, the incidence of unexpected uterine malignancies in hysterectomies carried out for benign indications was found to be 0.33%. Most UUM were at early stage and associated with abnormal bleeding. Therefore, preoperative examination in the context of benign hysterectomy must be undertaken with care, and patients should be educated about the very slight possibility of a malignant diagnosis. Abdominal hysterectomy with in-bag morcellation represented a time-efficient and feasible alternative to laparotomy, until better alternatives are discovered.
